# P-958. Evaluating Long-term Care Facility Antimicrobial Stewardship Programs with an Infection Control Assessment and Response Visit

**DOI:** 10.1093/ofid/ofaf695.1160

**Published:** 2026-01-11

**Authors:** Cullen Adre, Jasmine Watkins, Donna L Russell, Christopher D Evans, Christopher Wilson

**Affiliations:** Tennessee Department of Health, Nashville, Tennessee; Tennessee Department of Health, Nashville, Tennessee; Tennessee Department of Health, Nashville, Tennessee; Tennessee Department of Health, Nashville, Tennessee; Tennessee Department of Health, Nashville, Tennessee

## Abstract

**Background:**

Infection Control Assessment and Response (ICAR) visits utilize a series of CDC-developed modules to evaluate a healthcare facility’s infection prevention programs. The ICAR Module 10 focuses on a facility’s antimicrobial stewardship programs and core element achievement. ICAR visits are typically performed by infection preventionists (IPs) with varying degrees of antimicrobial stewardship assessment experience.

**Methods:**

The Tennessee Department of Health (TDH) developed an initiative to increase the uptake of preventative ICARs in long-term care facilities (LTCF). Due to the number of LTCFs requesting a module 10 review a TDH pharmacist developed training on stewardship assessments and developed follow-up questions and activity explanations. After the module 10 interview, IPs sent ICAR results and their feedback to the pharmacist for review. Feedback reports included summaries of programs’ strengths and weaknesses with suggestions and resources for improvement. Data were collected using REDCap electronic data capture tools hosted at TDH. All data analysis were completed using SAS Enterprise Guide 7.1.

**Results:**

Forty-five LTCFs participated from April–September 2024. The top stewardship activities that LTCFs implemented were having a stewardship policy (86.7%), assessing penicillin allergy (80%), and stopping unnecessary antibiotics in new *Clostridium difficile* infection cases (75.6%). Antibiotic use tracking was performed in 100% of LTCFs and most achieved by using electronic health data (86.7%). Individuals responsible for the management and outcomes of their facility’s stewardship program are IPs and directors of nursing (100%). Percent of time dedicated to stewardship was 50% or less in 89.7% of facilities.

**Conclusion:**

Antibiotic stewardship assessments at LTCFs using the ICAR Module 10 tool revealed that stewardship responsibilities primarily fall to IPs/DONs who often have their duties split between stewardship and other programs. Most facilities had at minimum a policy in place for antimicrobial stewardship, however, there remains room for improvement. This reinforces the need for additional resources and initiatives for LTCFs to improve stewardship and optimize antibiotic use.

**Disclosures:**

All Authors: No reported disclosures

